# Alterations in Organismal Physiology, Impaired Stress Resistance, and Accelerated Aging in *Drosophila* Flies Adapted to Multigenerational Proteome Instability

**DOI:** 10.1155/2019/7823285

**Published:** 2019-06-11

**Authors:** Maria S. Manola, Eleni N. Tsakiri, Ioannis P. Trougakos

**Affiliations:** Department of Cell Biology and Biophysics, Faculty of Biology, National & Kapodistrian University of Athens, 15784, Greece

## Abstract

Being an assembly of highly sophisticated protein machines, cells depend heavily on proteostatic modules functionality and on adequate supply of energetic molecules for maintaining proteome stability. Yet, our understanding of the adaptations induced by multigenerational proteotoxic stress is limited. We report here that multigenerational (>80 generations) proteotoxic stress in Oregon^R^ flies induced by constant exposure to developmentally nonlethal doses of the proteasome inhibitor bortezomib (BTZ) (*G80-BTZ* flies) increased proteome instability and redox imbalance, reduced fecundity and body size, and caused neuromuscular defects; it also accelerated aging. *G80-BTZ* flies were mildly resistant to increased doses of BTZ and showed no age-related loss of proteasome activity; these adaptations correlated with sustained upregulation of proteostatic modules, which however occurred at the cost of minimal responses to increased BTZ doses and increased susceptibility to various types of additional proteotoxic stress, namely, autophagy inhibition or thermal stress. Multigenerational proteome instability and redox imbalance also caused metabolic reprogramming being evidenced by altered mitochondrial biogenesis and suppressed insulin/IGF-like signaling (IIS) in *G80-BTZ* flies. The toxic effects of multigenerational proteome instability could be partially mitigated by a low-protein diet that extended *G80-BTZ* flies' longevity. Overall, persistent proteotoxic stress triggers a highly conserved adaptive metabolic response mediated by the IIS pathway, which reallocates resources from growth and longevity to somatic preservation and stress tolerance. Yet, these trade-off adaptations occur at the cost of accelerated aging and/or reduced tolerance to additional stress, illustrating the limited buffering capacity of survival pathways.

## 1. Introduction

Considering that cellular functionality is ensured by the highly wired action of sophisticated protein machines and that proteome instability (also referred to as proteotoxic stress) causes significant detrimental effects, it is not surprising that proteome homeodynamics (proteostasis) is central for cellular functionality and the overall healthspan of organisms [1]. To ensure proteostasis, cells have developed a network of modules to assist protein folding and counteract proteotoxic stress; this network is referred to as the proteostasis network (PN) [2]. PN ensures proteome quality control at both basal conditions and during conditions of proteome instability by addressing the triage decision of *fold*, *hold*, or *degrade* [3]. Key components of the PN are the protein synthesis and sorting/trafficking machineries, the molecular chaperones, and the two main degradation machineries, namely, the autophagy lysosome (ALP) and the ubiquitin proteasome (UPP) pathways [4, 5]. ALP is mainly involved in the degradation of damaged organelles and protein aggregates and consists of microautophagy, chaperone-mediated autophagy, and macroautophagy [6]. On the other hand, UPP ensures protein synthesis quality control and it degrades normal short-lived ubiquitinated proteins and nonrepairable misfolded or unfolded polypeptides [5]. Additional modules of the PN are considered to be the stress-responsive signaling pathways (e.g., heat or oxidative), including those of forkhead box O (Foxo) and nuclear factor erythroid 2-related factor (Nrf2) transcription factors. Nrf2 is involved in cell protection against xenobiotic or oxidative damage [7, 8], while Foxo regulates autophagic and metabolic responses [9, 10].

The 26S eukaryotic proteasome is a complex protein machine of ~2.5 MDa that comprises a 20S core particle (CP) bound to one or two 19S regulatory particles (RP) [11]. The 20S CP is composed of four stacked heptameric rings (two *α*-type surrounding two *β*-type rings) that form a barrel-like structure; the caspase- (C-L), trypsin- (T-L), and chymotrypsin- (CT-L) like peptidase activities are located at the *β*1, *β*2, and *β*5 proteasome subunits, respectively. Ubiquitinated polypeptides are degraded by the 26S proteasome [5, 12], while nonnative (e.g., oxidized) polypeptides are likely degraded by the 20S proteasome via chaperone-mediated targeting [13]. Furthermore, proteasome has been implicated in maintaining mitochondrial functionality [5] suggesting that proper function of proteostatic modules is also critical for cellular energetics and metabolism.

Yet, despite the tight PN regulation, a narrow boundary between health and disease exists, and subtle changes caused, for instance, by genetic mutations, environmental stress, or certain aspects of lifestyle (e.g., obesity) can trigger proteome instability. This process can then gradually overwhelm the PN leading to stress spreading within the same tissue and even across distinct organs by cell *nonautonomous* mechanisms [14]. Beyond these events, aging is undoubtedly the major risk factor for virtually all protein instability-related diseases. This correlation largely relates to reduced functionality of antistress responses and proteostatic modules during aging [15, 16]. Consistently, proteasomal dysfunction has been correlated with deregulation of the proteostasis network possibly underlying the early offset of aging phenotypes and aging-related diseases [5, 16].

Interestingly, aberrant activation of proteostatic modules marks the onset of carcinogenesis [17]; it is speculated that increased UPP activity during carcinogenesis is associated with tumor cell adaptation to elevated proteotoxic stress [1, 17]. Consistently, therapeutic targeting of the proteasome is currently used for the treatment of hematological malignancies and remains a challenge for the cure of solid tumors [18, 19]. UPP inhibitors which have demonstrated clinical efficacy include bortezomib (BTZ) [19] and carfilzomib [20]. BTZ is a slowly reversible inhibitor that binds the catalytic site of the 26S proteasome enabling inhibition of the CT-L and, to a lesser extent, of C-L and T-L activities [19, 21, 22]. Nevertheless, the development of severe adverse effects linked to the usage of proteasome inhibitors, such as peripheral neuropathies and/or cardiovascular diseases, along with inherent or acquired drug resistance remain a significant clinical problem [19, 23]. Acquired resistance to proteasome inhibition has been correlated in cellular models with UPP upregulation and/or mutations of the *PSMB5* gene; yet, no mutations have been found in the *PSMB5* gene in myeloma patients being refractory to or relapsed from BTZ therapy [24, 25].

By using *Drosophila* flies as a model organism to study cellular proteostasis in the young organism, during aging and in age-related diseases, we recently found that proteasome functionality is sex-, tissue-, and age-dependent [26]. Here, we report that multigenerational proteotoxic stress in Oregon^R^ flies induced by exposure to developmentally nonlethal doses of BTZ increased proteome instability and redox imbalance, reduced fecundity and body size, and caused neuromuscular defects; it also accelerated aging. Furthermore, multigenerational proteome instability caused metabolic reprogramming and increased susceptibility to various types of additional proteotoxic stress, namely, autophagy inhibition or thermal stress.

## 2. Materials and Methods

### 2.1. Fly Stocks and Treatment with BTZ

*G80-BTZ* flies were established by continuous treatment of Oregon^R^ flies with 0.5 *μ*Μ of the proteasome inhibitor bortezomib (BTZ) (PS-341) for more than 80 generations (>4 years). Flies were maintained at 23-24°C and 60% relative humidity on a 12 h light : 12 h dark cycle as described before [27] and were cultured in standard (SM), high protein (4x of the regular protein intake) (HPM), low protein (1/2 of the regular protein intake) (LPM), or low calories (CRM) medium (1x SM diluted 1x with boiled tap water).

### 2.2. Measurement of Growth and Developmental Effects: Climbing and Longevity Assays

For the effects of multigenerational proteotoxic stress on the developmental processes, five young female flies were placed in Petri dishes containing 1.5% agar dissolved in sour cherry juice and allowed to lay eggs for 24 h; embryos in each dish were then counted and collected using PBS. Forty embryos from each population were transferred in fresh standard medium and monitored for 14 days. Cultures were photographed daily to record growth rates and at the end of the assay, adult flies were counted and each developmental stage (larvae, early/late stage pupae, or adult flies) was captured using a stereomicroscope to probe possible defects during development and alternations in morphology, respectively.

Body size was examined by weighting young flies and by measuring the wings' perimeter. Twenty young female flies from each population were collected and allowed to lay eggs for 24 h; then, adult flies were removed, and embryos allowed to develop to adulthood. Middle-aged flies were collected in groups of ten individuals and starved for 1 h followed by weighting each group of flies to 0.01 mg accuracy with an analytical balance. Removed wings from middle-aged flies were placed on observation plates and captured using a digital camera adapted to a stereomicroscope, and measurement of the perimeter was performed using Image J (National Institutes of Health, USA).

Longevity and climbing performance were assayed as previously described [21]. For survival curves and statistical analysis, the Kaplan-Meier procedure and log-rank (Mantel-Cox) test were used; significance was accepted at *P* < 0.05. Statistical analyses for all presented longevity experiments are reported in Table S1.

### 2.3. Heat and Bang Sensitivity Assays

Heat and bang assays were performed as described before [28] with minor modifications. Briefly, two groups of fifteen sex-sorted young flies were collected and transferred separately in vials containing fresh medium. For the heat sensitivity assay, flies were incubated (the next day) for 10 min at 40°C in empty vials, whereas for the bang assay (it mostly reveals neuronal defects [29]), flies were placed in empty vials and were vortexed for 20 sec at room temperature (RT); in both assays, treated flies were allowed to recover at RT. Assays were videotaped, and the time of full paralysis (pause of purposeful movements) and recovery (retrieval of purposeful movements/climbing) were recorded.

Full methods and any associated references are available in Supplementary Materials and Methods.

## 3. Results

### 3.1. Multigenerational Developmentally Nonlethal Proteasome Inhibition in *Drosophila* Flies Increased Proteome Instability and Redox Imbalance: It Also Tended to Reduce Fecundity and Body Size, Caused Neuromuscular Defects, and Accelerated Aging

For this project, Oregon^R^ *D. melanogaster* flies were cultured continuously for more than 80 generations in the presence of 0.5 *μ*Μ BTZ (*G80-BTZ* flies); this concentration of BTZ was chosen since it does not induce detrimental mortality during development [30]. We also used *G80-BTZ* flies cultured in SM (*G80* flies) or in 1 *μ*M BTZ (*G80-BTZ*/1), as well as parental nontreated (*NT*) flies transiently exposed to 0.5 (*NT-BTZ*) or 1 *μ*M (*NT-BTZ*/1) BTZ (Fig.  S1).

As expected, *G80-BTZ* flies had lower proteasomal activities compared to *NT* flies (Fig.  S2A); also, *G80-BTZ* flies' tissues accumulated ubiquitinated (Fig.  S2B1) and carbonylated (Fig.  S2B2) polypeptides, as well as reactive oxygen species (ROS) (Fig.  S2C), indicating the existence (vs. *NT* flies) of extensive proteome instability and redox imbalance.

**Figure 1:**
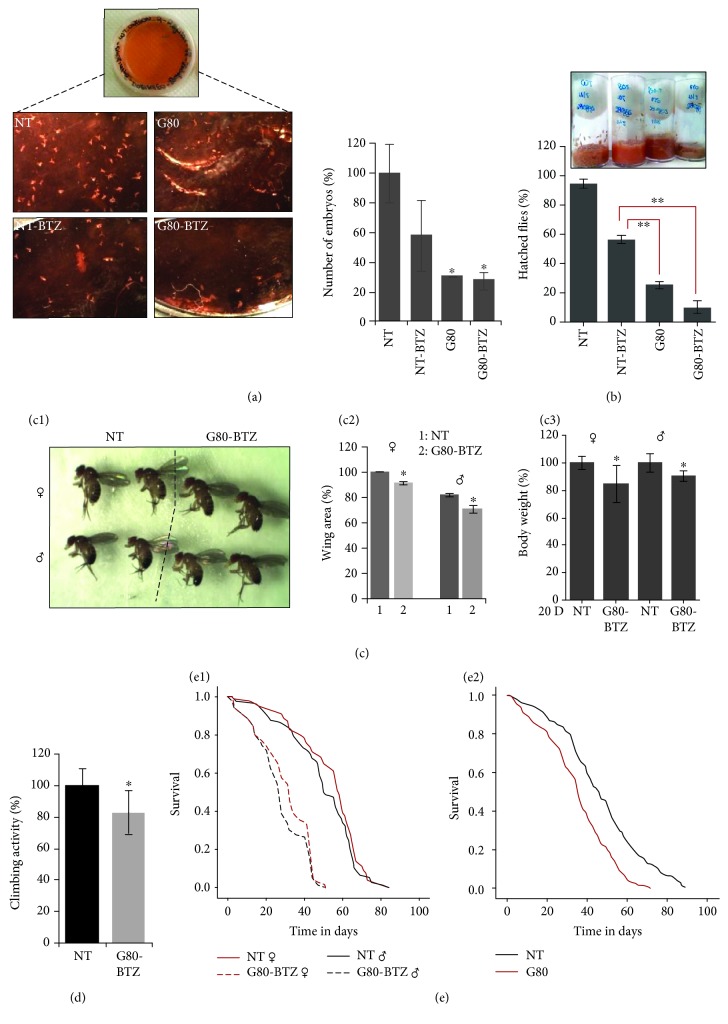
Multigenerational developmentally nonlethal proteasome inhibition in *Drosophila* flies reduced fecundity and body size, caused neuromuscular defects, and accelerated aging. (a) Laid embryos (%) during a period of 24 h by young *NT*, *NT-BTZ*, *G80*, and *G80-BTZ* females. (b) Hatched flies (%) 14 days posttransferring thirty embryos per population to the respective culture medium. (c1) Images of female and male flies of the *NT* and *G80-BTZ* groups. (c2) Area (%) of right and left wings dissected from young female or male flies of the *NT* and *G80-BTZ* populations. (c3) Body weight (%) of middle-aged female or male flies collected from the *NT* and the *G80-BTZ* groups. (d) Locomotion (climbing) activity of young *NT* and *G80-BTZ* flies. (e) Longevity curves of female and male *NT* and *G80-BTZ* flies (e1) or of *NT* and *G80* flies (e2); in (e2), equal numbers of female/male flies were used. Comparative statistics of the longevity assays are reported in Table S1. Bars, ±SD (*n* ≥ 2). ^∗^ *P* < 0.05; ^∗∗^ *P* < 0.01.

Our analyses also revealed that chronic proteotoxic stress decreased the rate of egg-laying (Figure 1(a)) and hatching of adult flies (Figure 1(b)) in *G80-BTZ* vs. *NT* flies; this effect was evident even when *G80-BTZ* flies were compared to *NT-BTZ* flies and was also retained in *G80* flies (Figures 1(a) and 1(b)), indicating permanent adaptations (a carry-over effect) in *G80-BTZ* flies' physiology and associated molecular responses. Furthermore, *G80-BTZ* flies showed sex-independent reduced (compared to *NT* flies) wing area (Figure 1(c1 and c2); indicative of body size) and body weight values (Figure 1(c3)) along with decreased neuromuscular activity (Figure 1(d)). *G80-BTZ* female and male flies had a significant reduction (vs. *NT* flies) in their longevity (Figure 1(e1); Table S1). This accelerated aging effect was also evident in *G80* flies (Figure 1(e2); Table S1) further supporting the notion of permanent alterations in flies' physiology and in reduced survival rates after multigenerational proteotoxic stress.

### 3.2. *G80-BTZ* Flies Were Mildly Resistant to BTZ and Showed No Age-Related Loss of Proteasome Activity; These Adaptations Likely Relate to Sustained Upregulation of Proteostatic Modules

**Figure 2:**
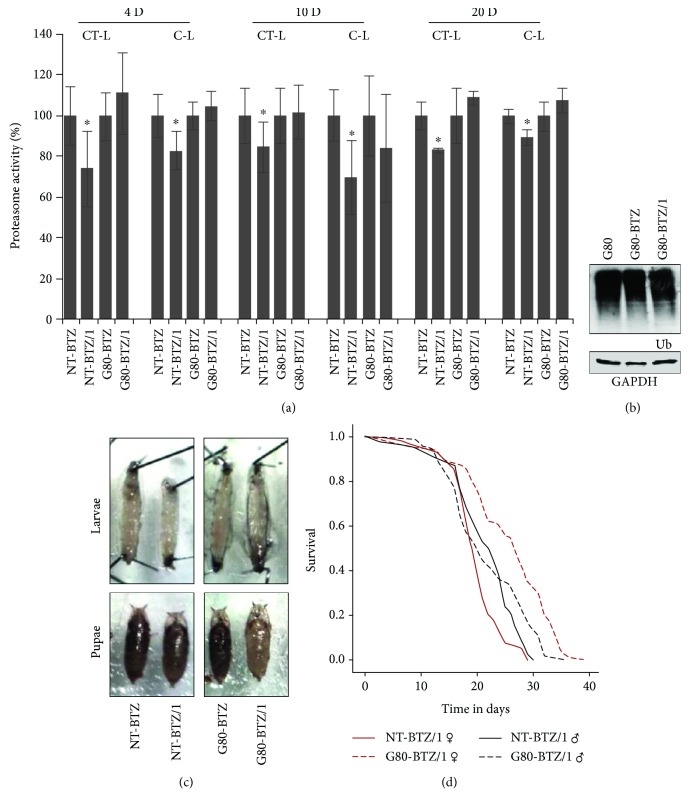
*G80-BTZ* flies were mildly resistant to BTZ. (a) Relative (%) CT-L and C-L proteasome activities in somatic tissues of *NT-BTZ* and *G80-BTZ* flies (see Fig.  S1) following exposure to 1 *μ*Μ *ΒΤZ* for 4 days (4D), 10 days (10D), or 20 days (20D). (b) Representative immunoblotting analysis of ubiquitin (Ub) levels in somatic tissue lysates of shown young flies' groups (compared to control samples from *NT* flies shown in Fig.  S2B1); GAPDH probing was used as input reference. (c) Stereoscopic images of 3rd instar larvae and late-stage pupae of indicated groups. (d) Longevity curves of female and male *NT-BTZ* and *G80-BTZ* flies exposed to 1 *μ*Μ ΒΤZ. Bars, ±SD (*n* ≥ 2). ^∗^ *P* < 0.05.

*G80-BTZ* flies have acquired a moderate resistance to increasing doses of BTZ on the contrary to *NT* flies, in which exposure to 0.5 and 1 *μ*M of the inhibitor promoted a gradual decrease of proteasomal activities; this effect was not evident in *G80-BTZ*/1 vs. *G80-BTZ* flies (Figure 2(a)). In support, *G80*, *G80-BTZ*, and *G80BTZ*/1 flies showed no major differences in the levels of somatic tissue proteome ubiquitination (Figure 2(b)). Morphological studies in larvae and pupae revealed that exposure to 1 *μ*Μ BTZ significantly reduced (compared to treatment with 0.5 *μ*Μ BTZ) the size of *NT-BTZ* larvae and pupae, whereas the size of *G80-BTZ* larvae and pupae remained virtually unaffected following exposure to 1 *μ*Μ BTZ (Figure 2(c)). In support, *G80-BTZ*/1 flies lived longer as compared to *NT-BTZ*/1 flies, with the most resistant being the *G80-BTZ*/1 female insects (Figure 2(d); Table S1). Notably, the acquired mild resistance to BTZ was not evident in higher BTZ doses (not shown).

Then, we asked whether the observed mild tolerance of *G80-BTZ* flies to higher BTZ doses is caused by alternations in the structure of the targeted proteasomes, which reduce the binding affinity of the inhibitor. By measuring the dose-dependent effect of BTZ on the activity of proteasomes isolated from *NT* and *G80-BTZ* flies, we found no differences in the level of inhibition (Fig.  S3A); thus, the hypothesis for proteasomal structural changes (e.g., due to acquired mutations) that might have triggered the mild resistance to BTZ was discarded. In support, prolonged culturing of *G80* flies in BTZ-free medium restored normal proteasomal peptidases' activities (Fig.  S3B), despite sustained proteome overubiquitination (Figure 2(b)) and redox imbalance (Fig.  S3C).

**Figure 3:**
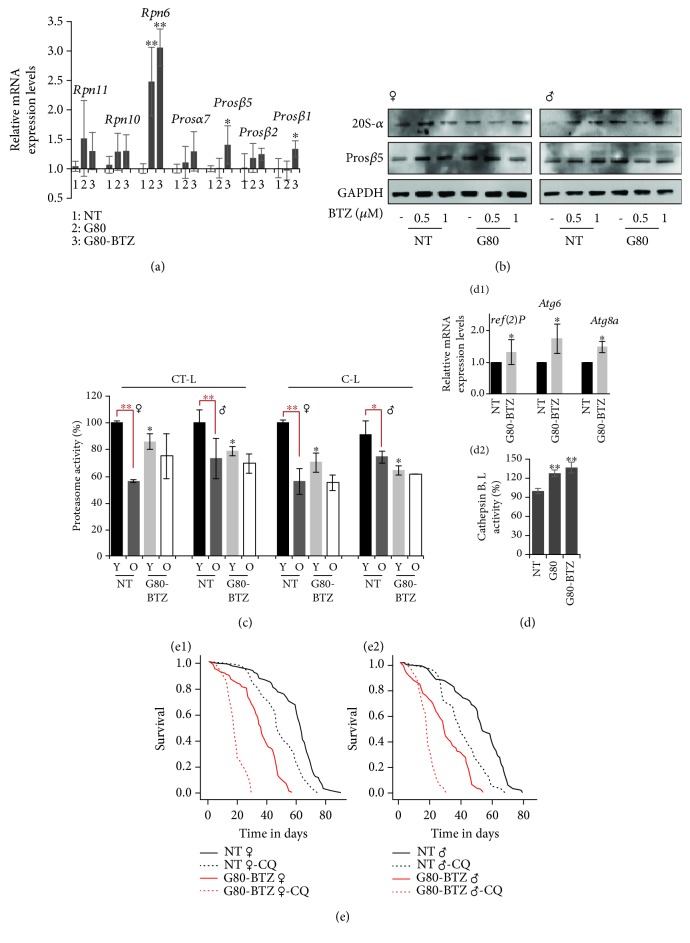
Multigenerational proteotoxic stress triggered the upregulation of proteostatic modules. (a) Relative expression of *Rpn11*, *Rpn10*, *Rpn6*, *Prosα7*, *Prosβ5*, *Prosβ2*, and *Prosβ1* proteasomal genes in somatic tissues of young *NT*, *G80*, and *G80-BTZ* flies; gene expression was plotted vs. the respective control (*NT* flies). (b) Representative immunoblotting analysis of 20S-*α* and Pros*β*5 proteasomal subunit expression levels in somatic tissues of young female and male *NT* and *G80* flies cultured (or not) in medium containing the indicated BTZ concentrations. (c) Relative (%) CT-L and C-L proteasome activities in young (Y) or aged (old; O) (≥80% of their lifespan) female/male flies of the shown groups. (d1) Relative expression of *ref(2)P*, *Atg6*, and *Atg8a* genes in somatic tissues of young *NT* and *G80-BTZ* flies; gene expression was plotted vs. the respective control *NT* flies. (d2) Relative (%) cathepsin B, L activity in somatic tissues of young *NT*, *G80*, and *G80-BTZ* flies. (e) Longevity curves of female (e1) and male (e2) flies of the indicated groups exposed to 200 *μ*Μ of the autophagy inhibitor chloroquine (CQ). The *Rp49* gene expression was used in (a), (b) as input reference. Bars, ±SD (*n* ≥ 2); ^∗^ *P* < 0.05; ^∗∗^ *P* < 0.01.

Multigenerational proteome instability promoted the induction (vs. *NT* flies) of 19S (*Rpn11*, *Rpn10*, and *Rpn6*) and 20S (*Prosα7*, *Prosβ5*, *Prosβ2*, and *Prosβ1*) proteasomal genes (Figure 3(a)) and of proteasomal protein subunits (20S-*α* and Pros*β*5) (Figure 3(b)) in *G80* and *G80-BTZ* flies. The induction of proteostatic modules was likely permanent, as *G80-BTZ* flies did not show any age-dependent reduction of proteasomal activities (Figure 3(c)), which is a hallmark of aging in *NT* flies [26]; this adaptation has likely occurred at the cost of reduced responsiveness (i.e., proteasomal subunit upregulation; [30]) to increased doses of BTZ (Figure 3(b)). Furthermore, young (Figure 3(d1)) and aged (Fig.  S4A) *G80-BTZ* flies expressed higher (compared to *NT*) levels of the autophagic *ref(2)P* (the fly ortholog of mammalian *SQSTM1/p62*), *Atg6*, and *Atg8a* genes; showed increased lysosomal staining in larva fat bodies (Fig.  S4B), and both the *G80* and *G80-BTZ* flies had upregulated activities of the lysosomal cathepsins B, L (Figure 3(d2)). Also, the *cathD* gene was induced (vs. *NT* flies) in *G80-BTZ* aged flies (Fig.  S4A). Consistently, the *G80-BTZ* flies were more sensitive (compared to *NT* flies) to chloroquine (CQ) (an autophagy inhibitor) (Figure 3(e)) indicating that *G80-BTZ* flies are increasingly dependent to ALP for survival.

Thus, multigenerational exposure to proteotoxic stress caused the upregulation of proteostatic modules at the cost of reduced responsiveness to increased stress.

### 3.3. Multigenerational Proteome Instability in *G80-BTZ* Flies Induced Metabolic Reprogramming Being Evidenced by Increased Mitochondrial Biogenesis and Suppressed Insulin/IGF-Like Signaling (IIS)

**Figure 4:**
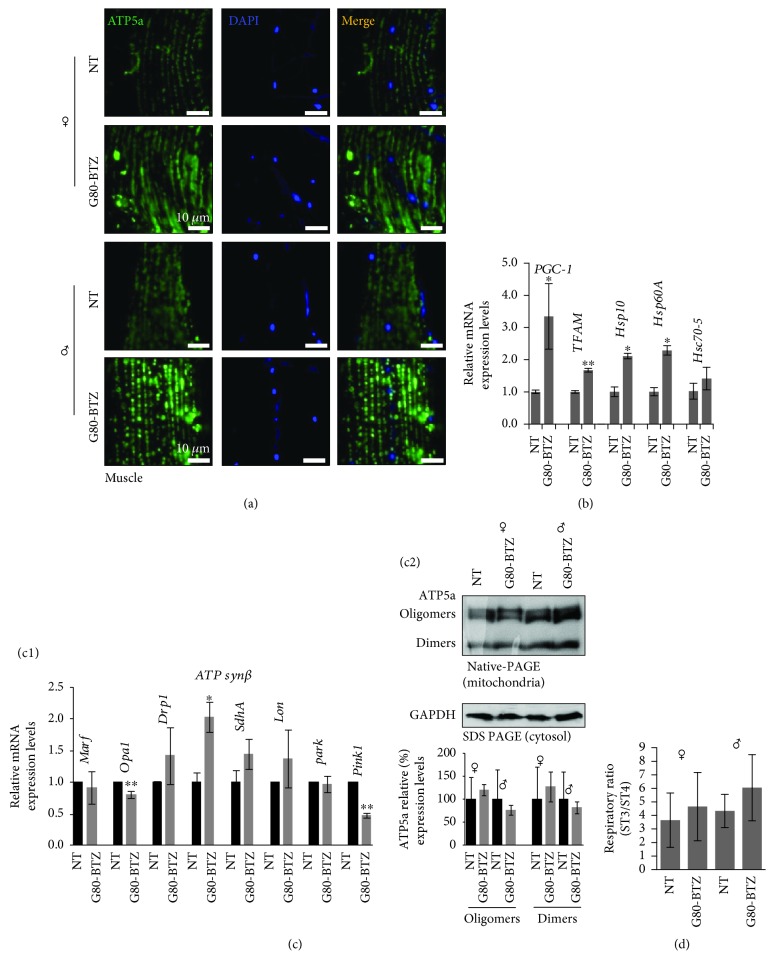
Sustained proteome instability caused metabolic reprogramming in *G80-BTZ* flies being evidenced by altered mitochondrial biogenesis. (a) CLSM visualization following immunofluorescence staining of young flies' thoracic muscle tissues with ATP5a antibody; samples were counterstained with DAPI. (b) Relative expression levels of mitochondrial biogenesis (*PGC-1*, *TFAM*) and chaperones (*Hsp10*, *Hp60A*, and *Hsc70-*5) genes in shown young flies' somatic tissues. (c1) Relative expression levels of mitochondrial energetics (*SdhA*, *ATPsynβ*), quality control (*Lon*), dynamics (*Marf*, *Opa1*, and *Drp1*), and mitophagy (*park*, *Pink1*) genes in indicated young flies' somatic tissues. (c2) ATP5a expression levels (blue native-PAGE; upper panel) and relative (%) quantitation (lower panel) in isolated mitochondria from somatic tissues of young female and male flies; GAPDH probing in cytosolic preparations was used as an input reference. (d) Mitochondrial ST3/ST4 respiratory ratio in somatic tissues of the indicated flies' groups. Gene expression was plotted vs. the respective control, and *Rp49* gene expression was used as input reference. Bars, ±SD (*n* ≥ 2). ^∗^ *P* < 0.05; ^∗∗^ *P* < 0.01.

Since IIS is implicated in the development of acquired resistance to BTZ in the clinic [31, 32], we then investigated whether the recorded adaptations in *G80-BTZ* flies were also associated with metabolic alterations. Confocal laser scanning microscopy (CLSM) analyses showed that multigenerational proteotoxic stress increased the number of mitochondria in the muscles of female and male *G80-BTZ* flies compared to *NT* (Figure 4(a)). This finding was in line with increased expression of the biogenesis-related mitochondrial genes *PGC-1* and *TFAM* (Figure 4(b)); we also noted the upregulation of mitochondrial chaperone genes (Figure 4(b)) consistent with reduced mitochondrial proteome carbonylation and ubiquitination (Fig.  S5). In addition, *G80-BTZ* flies showed reduced expression levels of the *Opa1* (involved in fusion of mitochondrial inner membrane, cristae remodeling, and energetics regulation) and *Pink1* (mitophagy-related) genes and expressed higher levels of the *ATPsynβ* gene (Figure 4(c1)); also, *G80-BTZ* females tend (not reaching statistical significance) to express higher levels of the ATP synthase dimers and oligomers (Figure 4(c2)). In support, *G80-BTZ* flies showed a trend (not statistically significant) towards increased mitochondrial respiration rates (Figure 4(d)). Thus, chronic proteotoxicity affected mitostatic pathways.

**Figure 5:**
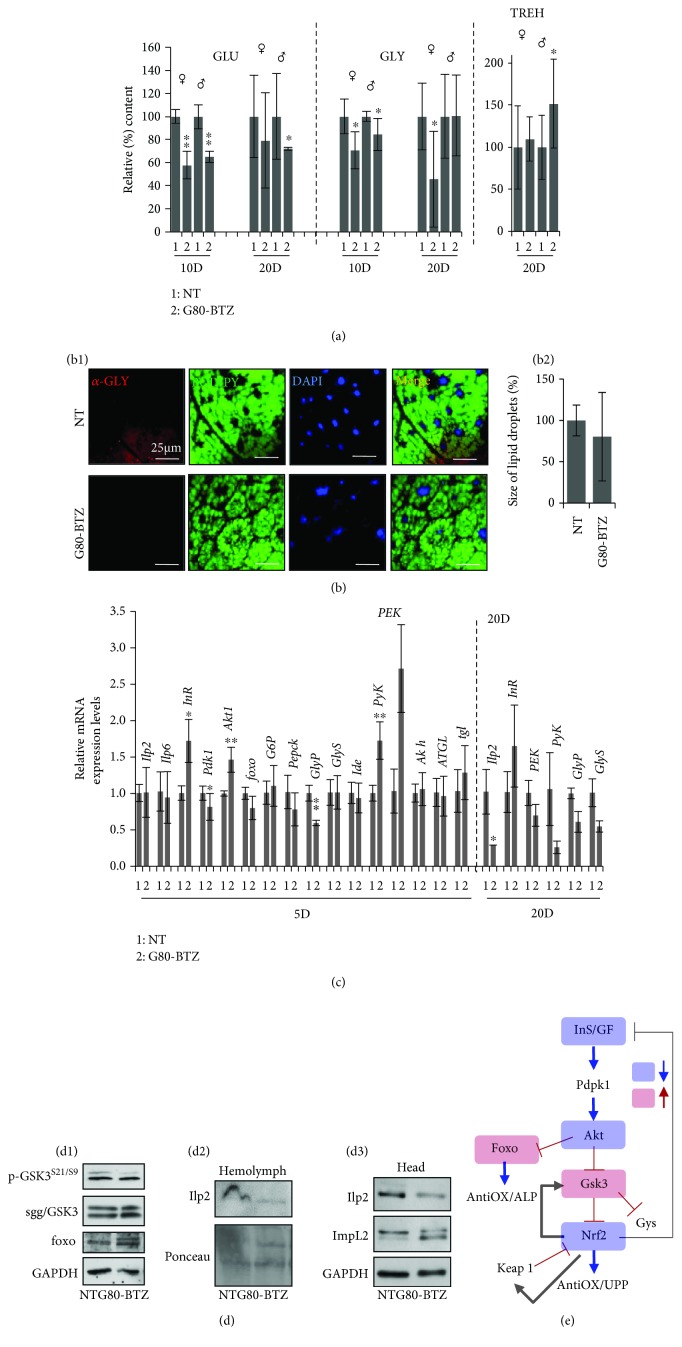
Multigenerational proteome instability in *G80-BTZ* flies induced metabolic reprogramming being evidenced by suppressed insulin/IGF-like signaling (IIS). (a) Relative (%) content of glucose (GLU), glycogen (GLY), and trehalose (TREH) levels in somatic tissues of female/male flies (10 or 20 days old) of the *NT* and *G80-BTZ* groups. (b1) CSLM visualization of fat bodies' (microdissected from 20-22-day-old female flies of the *NT* and *G80-BTZ* groups) lipid content after BODIPY staining; samples were also stained with a GLY antibody and counterstained with DAPI. (b2) Relative (%) size of lipid droplets shown in (b1). (c) Relative expression of *Ilp2*, *Ilp6*, *InR*, *Pdk1*, *Akt1*, *foxo*, *G6P*, *Pepck*, *GlyP*, *GlyS*, *Ide*, *PyK*, *PEK*, *Akh*, *ATGL*, and *tgl* genes in somatic tissues of flies of the *NT* and *G80-BTZ* populations. Gene expression was plotted vs. the respective control; the *Rp49* gene expression was used as input reference. (d) Immunoblotting analysis of protein expression in female somatic tissues (d1), haemolymph (d2), or dissected heads (d3) of *NT* and *G80-BTZ* flies; blots were probed with antibodies against p-GSK3^S21/S9^, sgg/GSK3, and foxo (d1); Ilp2 (d2); and Ilp2 and ImpL2 (d3). GAPDH or Ponceau S staining was used as loading reference. (e) Schematic representation of the IIS regulatory pathway in the context of Nrf2 and Foxo regulation. Bars, ±SD (*n* ≥ 2). ^∗^ *P* < 0.05; ^∗∗^ *P* < 0.01.

As mitochondrial function is tightly related to metabolic pathways' regulation, we also studied sugar and lipid metabolism in *G80-BTZ* flies' tissues. We found reduced (vs. *NT* flies) levels of glucose (GLU) and glycogen (GLY), along with a tendency (not statistically significant) for increased Trehalose (TREH) levels in male flies (Figure 5(a)) indicating a likely hyperglycemic state; TREH is the circulating sugar in flies' haemolymph being synthesized in the fat body (the equivalent of mammalian liver and adipose tissue in flies). In support, we noted reduced GLY staining in the fat body of adult flies and a tendency for increased lipolysis in *G80-BTZ* flies' fat body (Figure 5(b)). The noted metabolic adaptations were also evident at the genomic level as we found the upregulation of the *Akt1*, *PyK* (involved in glycolysis), and *InR* (insulin-like receptor) genes, along with the downregulation of the *Pdk1* (involved in Krebs cycle regulation) and *GlyP* (promotes GLY degradation) genes in *G80-BTZ* vs. *NT* young flies; in middle-aged flies, we also observed the suppression of the *Ilp2* gene (Figure 5(c)). Furthermore, we observed (mostly in female *G80-BTZ* flies) reduced expression of the inhibitory S^21^/S^9^ phosphorylated form of sgg/GSK3 (shaggy, the fly ortholog of mammalian glycogen synthase kinase-3) and increased *foxo* expression (Figure 5(d1)). We also observed the downregulation of the Ilp2 protein (ortholog to mammalian insulin; secreted from insulin-producing brain cells by cell-autonomous GLU sensing) in both the haemolymph (Figure 5(d2)) and the head tissue (Figure 5(d3)) of *G80-BTZ* flies; consistently, the *Ilp2* gene was downregulated in *G80-BTZ* flies (Figure 5(c)). Thus, multigenerational proteotoxic stress suppressed IIS (Figure 5(e)). In line with these findings, ImpL2 (a muscle-secreted factor that inhibits Ilp2 activity) expression levels were elevated in *G80-BTZ* flies (Figure 5(d3)) and thus, the observed upregulation of *InR* and *Akt1* genes (Figure 5(c)) is likely a late compensatory response due to suppressed IIS.

Taken together, these findings suggest that chronic proteome instability triggered metabolic reprogramming that suppressed IIS in adapted flies, indicating that prolonged stress signaling reallocates resources from growth and longevity to somatic preservation and stress tolerance.

### 3.4. Multigenerational Proteotoxic Stress-Mediated Toxicity Can Be Partially Mitigated by a Low-Protein Diet; yet, *G80-BTZ* Flies Were Increasingly Sensitive to Thermal Stress

**Figure 6:**
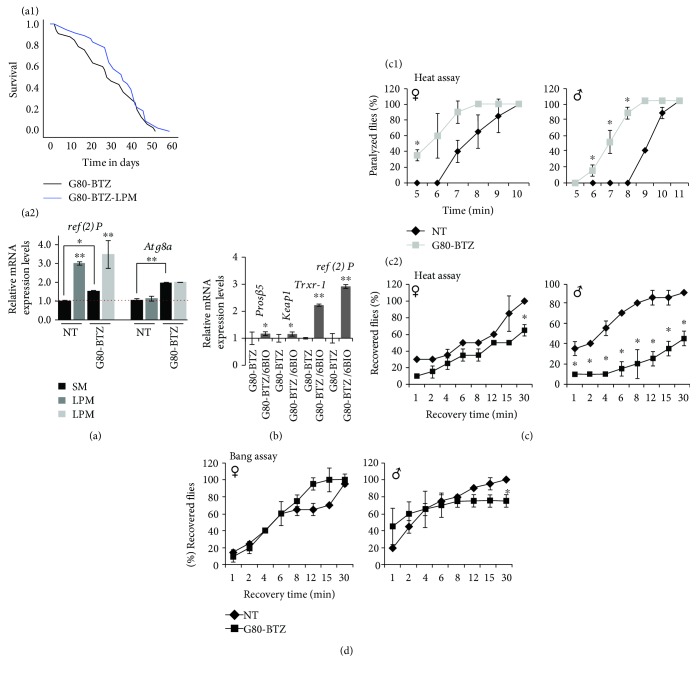
The toxicity of multigenerational proteotoxic stress can be partially mitigated by a low-protein-content diet; *G80-BTZ* flies were sensitive to thermal stress. (a1) Longevity curves of *G80-BTZ* flies fed (or not) with low-protein-content medium (LPM). (a2) Relative expression of the *ref(2)P* and *Atg8a* genes in *NT* or *G80-BTZ* flies fed (or not) with LPM. (b) Relative gene expression of proteasomal (*Prosβ*5, *Keap1*), antioxidant (*Trxr-1*), and autophagy-related (*ref(2)P*) cncC/Nrf2 transcriptional targets in somatic tissues of young *G80-BTZ* flies cultured for 5 days in medium containing (or not) 400 *μ*Μ 6BIO. (c) Recorded (%) female or male paralyzed flies (c1) following exposure for 10 min to 40°C and rate (%) of recovery (c2) at room temperature. (d) Rate of flies' recovery (%) after vortexing (bang assay) for 20 seconds. Gene expression was plotted vs. the respective control and *rp49* gene expression was used as input reference. Bars, ±SD (*n* ≥ 2). ^∗^ *P* < 0.05; ^∗∗^ *P* < 0.01.

IIS suppression is likely a prosurvival adaptation in *G80-BTZ* flies, which (among others) culminates in ALP activation (Figures 3(d), S4; see also, Figure 5(e)), a pathway shown before to correlate with increased longevity [16]. In support, exposure of *G80-BTZ* flies to a low-protein-content diet tended (not reaching statistical significance) to extend the median longevity of *G80-BTZ* flies (Figure 6(a1); Table S1). A high-protein or a low-calories diet was toxic in *G80-BTZ* flies (Fig.  S6), while no significant effects on longevity were found in parental *NT* or *NT-BTZ* flies fed with low-protein-content diet (Fig.  S7). Interestingly, apart from the acquired differences in the basal expression levels of the autophagic *ref(2)P* and *Atg8a* genes, low-protein-content diet did not induce significantly distinct responses in *ref(2)P* and *Atg8a* genes in the *NT* and *G80-BTZ* flies' populations (Figure 6(a2)) indicating that other pathways likely contribute to increased longevity of low-protein diet-fed *G80-BTZ* flies.

The acquired sustained suppression of IIS in *G80-BTZ* flies would also result in decreased activity of cytoprotective Nrf2 (Figure 5(e)). Indeed, treatment of *G80-BTZ* flies with 6-bromo-indirubin-3′-oxime (6BIO, a selective sgg/GSK3 inhibitor) [33] resulted in increased expression levels of cncC (the fly ortholog of mammalian Nrf2) transcriptional targets (Figure 6(b)). This observation would then indicate enhanced sensitivity of *G80-BTZ* flies to additional and/or to other types of proteome instability-inducing stress factors. Consistently, *G80-BTZ* flies were increasingly susceptible in a sex-independent manner to thermal stress as they were paralyzed faster (Figure 6(c1)) and recovered with a slower rate (Figure 6(c2)) vs. *NT* flies after exposure to heat shock, whereas they were unaffected by mechanical stress (Figure 6(d)).

Therefore, the trade-off adaptations under conditions of chronic proteotoxic stress occur at the cost of reduced tolerance to additional proteotoxic stress illustrating the limited buffering capacity of higher metazoan survival pathways.

## 4. Discussion

As acquired resistance to BTZ is often seen in the clinic in treated myeloma patients [34], and proteinopathies (such as neurodegenerative disorders) show high prevalence due to increased aging of the population [2], gaining insights into the molecular adaptations triggered at the organismal level due to chronic proteome instability is paramount for the development of complementary or alternative treatments for these devastating diseases. By developing the first *in vivo* model of multigenerational (>80 generations/>4 years) developmentally nonlethal proteome instability (also accompanied by redox imbalance) in *Drosophila* flies, we aimed to understand how proteotoxic stress acts on the evolutionary dynamics of populations and shapes stress response(s) across generations. Our findings indicate that chronic proteotoxic stress triggers a series of adaptations on flies' physiology including reduced fecundity and body size, locomotion defects, and accelerated aging. Similarly, to our findings, exposure of *Caenorhabditis elegans* to low concentration of bisphenol A (an organic synthetic compound exhibiting estrogen-mimicking properties) across four generations resulted in individuals that grew smaller, moved slower, and produced less offspring as compared to controls [35]. Also, exposure of *Daphnia magna* to elevated temperature for three generations significantly decreased the offspring's number, the time to first brood, and the body length compared to animals grown under optimal temperature; these effects were accompanied by increased ROS and lipid peroxidation [36].

Although studies in nonmyeloma or myeloma cell lines have suggested that acquired resistance enhanced proteasome activity and/or promoted mutations in the CT-L-related *β*5 proteasomal subunit that impaired BTZ binding [19, 37]; mutations have not been detected in the *PSMB5* gene of multiple myeloma (MM) patients being refractory to or relapsed from BTZ treatment [24, 25]. In support, we found no impairment of BTZ binding in isolated proteasomes from *G80-BTZ* flies, in which the acquired mild resistance to BTZ likely relates to sustained upregulation of proteostatic modules; these adaptations were largely evident in *G80* flies suggesting a carry-over effect. Indeed, it has been found that upon reversion to standard nutrition, flies whose prior generations have been exposed to a high-protein diet displayed multigenerational inheritance of altered gene expression [38]. Moreover, populations of *Drosophila subobscura* retain signatures from past contamination events with heavy metals [39], while exposure to G418 stress reduces the maternal levels of polycomb in the offspring embryos; this reduction contributed to the inheritance of induced gene expression patterns [40].

In parallel, we noted increased activities of lysosomal cathepsins and autophagic genes which along with increased sensitivity of *G80-BTZ* flies to CQ suggested that they gradually became dependent for their survival to higher ALP activities. Consistently, inhibition of ALP with CQ potentiates carfilzomib-induced apoptosis in myeloma cells *in vitro* and *in vivo* [41]. Also, in accordance with our findings, other reported mechanisms of acquired resistance to BTZ involve reprogramming of the Nrf2 pathway or IIS and/or upregulation of the heat shock response signaling pathway [19].

These prosurvival adaptations in *G80-BTZ* flies likely occurred at the cost of minimal responses of proteostatic modules to increased BTZ doses and increased susceptibility to various types of additional proteotoxic stress, namely, autophagy inhibition or thermal stress. Consistently, it was found that recurrent stress across three generations of female rats may cumulatively increase stress vulnerability and the risk of adverse health outcomes through perinatal programming [42]. In general, it is assumed that the noted adaptations relate to epigenetic effects; for instance, DNA hypomethylation of inflammation-associated genes in the adipose tissue has been described as an effect of multigenerational high-protein feeding in female mice [43], while increased expression of Hsp70 accompanied by changes in histone H3 methylation and histone H4 acetylation has been observed in *Artemia salina* (brine shrimp) after exposure to thermal stress [44]. Similarly, it was proposed that the evolution of insecticide resistance results from epigenetic modifications, which are heritable and influence gene expression without changing the underlying DNA sequence [45].

Interestingly, multigenerational proteome instability and redox imbalance in *G80-BTZ* flies caused metabolic reprogramming being evidenced by increased mitochondrial biogenesis and suppressed insulin/IGF-like signaling (IIS); consistently, we recently found that transient proteasome dysfunction disrupted mitochondrial morphology and function [8, 46]. Altered mental health through metabolic pathways' reprogramming has been documented after exposure of four generations of rats during pregnancy to stress [47]. Beyond energetics, mitochondria are also central executors of apoptosis; thus, altered mitochondrial function could infer changes to apoptosis process. Indeed, BTZ-resistant cells had mitochondrial adaptations that minimized induction of apoptosis [48], while induction of mitochondrial biogenesis enhanced resistance to several apoptotic stimuli in myocytes [49].

Prolonged stress signaling seems to be centrally linked to IIS downregulation since genotoxic stress in XPF-ERCC1-deficient mice reallocates resources from growth to the somatic preservation and life extension [50]; similarly, multigenerational proteotoxic stress induced, mainly in female flies, the downregulation of IIS. It has been reported that IIS is implicated in the development of acquired resistance to BTZ since IGF-1 enhanced the cytotoxic effect of proteasome inhibitors [32]. Therefore, IIS suppression is likely a prosurvival adaptation in *G80-BTZ* flies, which (among others) activated ALP, a pathway shown before to correlate with increased longevity [16]; thus, it is not surprising that exposure of *G80-BTZ* flies to low-protein diet tends to increase their median longevity.

Overall, our presented data highlight the toxic effects of multigenerational proteotoxic stress and the extensive functional wiring of proteostatic and metabolic/energetic pathways, indicating also that higher metazoans maximize fitness by adopting prosurvival alterations in proteostatic-mitostatic-metabolic pathways in response to prolonged proteome instability. Part of these adaptations to chronic proteotoxic stress includes a highly conserved adaptive metabolic response mediated by the IIS pathway, which reallocates resources from growth and longevity to somatic preservation and stress tolerance. Yet, these constraints and trade-off adaptations take place at the cost of accelerated aging and/or reduced tolerance to additional stress illustrating the exhaustion of the survival pathway buffering capacity.

## Data Availability

The datasets generated and/or analyzed during the current study are available from the corresponding author on reasonable request.

## Supplementary Materials

Supplementary Materials Supplementary Information includes figures and associated legends; Table S1 (longevity assay statistics); list of abbreviations; Materials and Methods; and associated References.
