# Supplementary material for: Alterations in Organismal Physiology, Impaired Stress Resistance, and Accelerated Aging in Drosophila Flies Adapted to Multigenerational Proteome Instability
Source: Oxid Med Cell Longev. 2019 Jun 11;2019:7823285. doi: 10.1155/2019/7823285 (PMC6610734; doi:10.1155/2019/7823285)
Supplement: Supplementary Materials — Supplementary Information includes figures and associated legends; Table S1 (longevity assay statistics); list of abbreviations; Materials and Methods; and associated References. [file 7823285.f1.pdf]

Alterations in organismal physiology, impaired stress resistance, and accelerated aging in *Drosophila* flies adapted to multigenerational proteome instability

Maria S. Manola, Eleni N. Tsakiri and Ioannis P. Trougakos

Supplementary Information

Supplementary Figures Legends

Figure S1. Drawing showing the different culture conditions and corresponding nomenclature of non-treated (*NT*) Oregon^R (A) and of *G80-BTZ* (exposed for >80 generations to 0.5 μM BTZ) (B) flies. *NT* or *G80-BTZ* flies were transiently exposed to standard medium (SM) or to SM containing either 0.5 or 1 μM BTZ as indicated.

Figure S2. Multigenerational developmentally non-lethal proteasome inhibition in *Drosophila* flies increased proteome instability and redox imbalance. (A_1) Relative (%) CT-L and C-L proteasome activities in young *G80-BTZ* female and male flies' somatic tissues lysates; comparisons were *vs*. proteasome activities of *NT* flies. (A_2) Analysis of isolated 26S proteasomes from somatic tissues of young *NT* and *G80-BTZ* flies under ultraviolet light (UV) and in Native-PAGE following probing with a Prosβ5 antibody. (B) Immunoblot analysis of proteome ubiquitination (Ub) (B_1) and carbonylation (dinitrophenol/DNP) (B_2) in somatic tissues of young *NT vs*. *G80-BTZ* flies. GAPDH probing was used in (A_2, B_1, B_2) as input reference. (C) (%) ROS levels in shown populations of middle-aged flies. Bars, ± SD (n=2); * $P < 0.05$; ** $P < 0.01$.

Figure S3. Analyses of proteasome activities after adding BTZ in lysates containing intact proteasomes or in shown flies' tissues; and tissue ROS levels. (A) Relative (%) CT-L and C-L peptidase activities following addition of the shown BTZ concentrations in flies' somatic tissue lysates containing intact proteasomes. (B) Relative (%) CT-L and C-L proteasome activities in somatic tissues of *NT*, *G80* and *G80-BTZ* flies at the age of 4, 10, 20 or 30 days. (C) (%) ROS levels in somatic tissues of the indicated middle-aged flies. Bars, ± SD (n=2). * $P < 0.05$; ** $P < 0.01$.

Figure S4. Autophagic genes expression in *NT* and *G80-BTZ* aged flies and LysoTracker™ staining in control and *G80-BTZ* larvae fat bodies. (A) Relative expression of *ref(2)P*, *Atg6*, *Atg8a* and *cathD*

genes in somatic tissues of aged *NT* and *G80-BTZ* flies; gene expression was plotted *vs*. the respective control (*NT* flies). (B_1) Staining with LysoTracker^{TM} of isolated fat bodies from *NT* or *G80-BTZ* larvae and (B_2) relative (%) lysosome quantification per μm^2, (n) nucleus. The *Rp49* gene expression was used in (A) as input reference. Bars, ± SD (n=2); * $P < 0.05$.

Figure S5. Proteome carbonylation and ubiquitination in mitochondria isolated from *NT* and *G80-BTZ* adult flies. Blots were probed with antibodies against DNP (carbonylation) and Ub; ATP5a and GAPDH were used a loading references in mitochondria and cytosol samples respectively.

Figure S6. The effect of high protein or low calories diet on *G80-BTZ* flies. Longevity curves of *G80-BTZ* flies cultured in high protein (A) (HPM) or low calories (B) (CRM) medium. Equal numbers of female/male flies were used in each experiment. Statistics of the longevity assays are reported in Table S1; the shown *G80-BTZ* longevity curve is identical in (A), (B).

Figure S7. The effect of low protein diet on *NT* flies treated (or not) with 0.5 μM BTZ. Longevity curves (*vs*. controls) of *NT* flies exposed (B) [or not (A)] to 0.5 μM BTZ and co-cultured in low protein medium (LPM). Equal numbers of female/male flies were used in each experiment; comparative statistics of the longevity assays are reported in Table S1.

Graphical Abstract. Our summarized findings indicate that multigenerational proteotoxic stress and redox imbalance causes metabolic reprogramming and impaired stress resistance; it also promotes fecundity and neuromuscular defects, and accelerates aging.

Supplementary Tables

Table S1. Summary of lifespan experiments.

Supplementary list of Abbreviations

19S regulatory particle, RP; 20S core particle, CP; 6-bromo-indirubin-3′-oxime, 6BIO; Adenosine triphosphate, ATP; Autophagy-lysosome pathway, ALP; Bortezomib, BTZ; Caspase-like activity, C-L; Chymotrypsin-like activity, CT-L; Forkhead box O, Foxo; Glucose, GLU; Glycogen synthase kinase, Gsk3; Glycogen, GLY; Heat shock protein, Hsp; Insulin/IGF- like signaling, IIS; Multiple myeloma, MM; Nuclear factor erythroid 2-related factor, Nrf2; Non-treated, NT; Proteostasis network, PN; Reactive oxygen species, ROS; Standard medium, SM; Trehalose, TREH; Ubiquitin, Ub; Ubiquitin-proteasome pathway, UPP.

KEY RESOURCES TABLES

REAGENT OR RESOURCE	SOURCE	IDENTIFIER
Antibodies		
Prosβ5 (β5)	M. Figueiredo-Pereira	Vernace et al. 2007
26S proteasome α (IIG7) (20Sα)	Santa Cruz Biotechnology	sc-65755
Complex V subunit-ATP5a	Abcam	ab14748
Ilp2	Prof. Ernst Hafen	N/A
Foxo	Cosmo Bio Co	CAC-THU-A-FOXO
ref(2)P (p62)	Prof. Gábor Juhász University,	N/A
sgg/GSK-3β	Millipore	05-412
ImpL2	Prof. Ernst Hafen	N/A
p-GSK3 α/β $Ser^{21/9}$	Cell Signalling	9331S
α-GLY	Prof. O. Baba	N/A
Ubiquitin	Santa Cruz Biotechnology	sc-8017
GAPDH	Sigma-Aldrich	G9545
HRP-conjugated anti-mouse IgG	Santa Cruz Biotechnology	sc-2005
HRP-conjugated anti-goat IgG	Santa Cruz Biotechnology	sc-2020
HRP-conjugated anti-rabbit IgG	Santa Cruz Biotechnology	sc-2004
Anti-Rabbit-IgG AlexaFlour 647	Jackson ImmunoResearch	711-605-152
Anti-Mouse-IgG AlexaFlour Plus 555	Jackson ImmunoResearch	A327327
Chemicals, Peptides		
16 % formaldehyde	Polysciences	18814-20
2-mercaptoethanol	Merck	15433
5 % Digitonin	Thermo Fischer Scientific	BN2006
6-bromo-indirubin-3'-oxime (6BIO)	Prof. A.L. Skaltsounis	N/A
Acrylamide	Sigma-Aldrich	A3574
ADP	Sigma	A2754
Agar	Fluka	5040
Amyloglucosidase	Sigma-Aldrich	A7420
APS	Research Organics	9530A
ATP	Sigma-Aldrich	1905
BSA	Sigma-Aldrich	A9418
CH_3COOH	Merck	K39105963

Chloroquine	Sigma-Aldrich	C6628
$ClCH_2COONa$	Fluka	24610
Coomasie Blue	Fluka	27816
Developer D-19	Kodak	146 4593
Diethyl malate	Sigma-Aldrich	W237418
DMSO	Sigma	270431
DTT	Applichem	A2948
EDTA	Applichem	A2937
EGTA	Applichem	A0878
EtOH	Sigma-Aldrich	46139
FCCP	CAYMAN CHEMICAL	370-86-5
Fixer	Agfa	G382B
Glutamic acid	Sigma-Aldrich	G1251
Glycerol	Applichem	A2926
Glycine	Serva	23390
Hepes	Biosera	PM-B2093
KCl	Applichem	131494
KH_2PO_4	Applichem	A1043
MeOH	Scharlau	ME0316005l
MG-132	Enzo Life Sciences	260-092-M05
$MgCl_2$	Sigma-Aldrich	13152
Mowiol®	Sigma-Aldrich	4-88
Glutamic acid	Sigma-Aldrich	G1251
Na_2HPO_4	Sigma-Aldrich	S3264
NaCl	Merck	1.06405.1000
Native-PAGE sample additive G250 5%	Invitrogen	BN2004
Native-PAGE sample buffer	Invitrogen	BN20032
NP-40	Sigma	NP40S
Oligomycin	Sigma-Aldrich	75351
PBS	Sigma	P4417
PIC (proteases inhibitor cocktail)	Sigma	I5763
Propionic acid	Sigma-Aldrich	402907
SDS	Serva	20765

Suc-Leu-Leu-Val-Tyr-AMC-LLVY	Enzo Life Sciences	BML-P802-0005
Sucrose	Applichem	A2211
TEMED	Applichem	A1148
Trehalase	Sigma-Aldrich	T8778
Tricine	Sigma-Aldrich	5704-04-1
Tris-HCl	Applichem	A3452
Triton X-100	Applichem	A4975
Tween 20	Sigma	P-1379
z-FR-AMC	Enzo Life Sciences	BML-P139-0050
Z-Leu-Leu-Glu-AMC-LLE	Enzo Life Sciences	BML-ZW9345-0005
Critical Commercial Assays/Kits		
Bradford assay	Bio-Rad	5000006
GLU Reagent	Sigma-Aldrich	GAGO-20
Onescript cDNA synthesis kit	ABMGOOD	G233
HOT FIREPol Eva green qPCR Mix Plus	SolisBioDyne	08-24-00001
Western ECL Blotting substrates	Bio-Rad	1705060
Native-PAGE 3-12% gradient pre-cast Bis-Tris gels	Novex-Life Technologies	BN1001BOX
Nitrocellulose membrane	Macherey-Nagel GmbH	741280
PVDF membrane	Macherey-Nagel GmbH	741290
OxyBlot	Millipore	#s7150
Fluorescent dyes		
BODIPY 493/503	Molecular Probes™ - TFS	D3922
LysoTracker™ Deep Red	Invitrogen	L12492
DAPI	Molecular Probes™ - TFS	D1306
Oligonucleotides		
See Method Details for primer sequences	This study	N/A
Software and Algorithms		
MS Excel	Microsoft	N/A
IBM SPSS; version 24.0	IBM	N/A
ImageJ	Wayne Rasband (NIH)	N/A
Digital Eclipse Nikon C1 software	Nikon Inc.	N/A

Supplementary Materials and Methods

List of Genes

Rpn11 (Regulatory particle non-ATPase 11, FBgn0028694, CG18174); *Rpn10* (Regulatory particle non-ATPase 10, FBgn0015283, CG7619); *Rpn6* (Regulatory particle non-ATPase 6, FBgn0028689, CG10149); *Prosα7* (Proteasome α7 subunit, FBgn0023175, CG1519); *Prosβ5* (Proteasome β5 subunit, FBgn0029134, CG12323); *Prosβ2* (Proteasome β2 subunit, FBgn0023174, CG3329); *Prosβ1* (Proteasome β1 subunit, FBgn0010590, CG8392); *ref(2)P* (refractory to sigma P-RNA and export factor binding protein 2, FBgn0003231, CG10360); *Atg6* (Autophagy-related 6, FBgn0264325, CG5429); *Atg8a* (Autophagy-related 8a, FBgn0052672, CG32672); *cathD* (cathepsin D, FBgn0029093, CG1548); *PGC-1* (*srl*, spargel, FBgn0037248, CG9809); *TFAM* (mitochondrial transcription factor A, FBgn0038805, CG4217); *CG11267* (also known as *Hsp10*, 10kDa heat shock factor, mitochondria-like, FBgn0036334, CG11267); *Hsp60A* (Heat shock protein 60A, FBgn0015245, CG12101); *Hsc70-5* (Heat shock 70 kDa protein cognate 5, FBgn0001220, CG8542); *Marf* (Mitochondrial assembly regulatory factor, FBgn0029870, CG3869); *Opa1* (Optic atrophy 1, FBgn0261276, CG8479); *Drp1* (Dynamin related protein 1, FBgn0026479, CG3210); *ATPsynB* (ATP synthase, subunit B, FBgn0019644, CG8189); *SdhA* (Succinate dehydrogenase, subunit A, flavoprotein, FBgn0261439, CG17246); *Lon* (Lon protease, FBgn0036892, CG8798); *park* (parkin, FBgn0041100, CG10523); *Pink1* (PTEN-induced putative kinase 1, FBgn0029891, CG4523); *Ilp2* (Insulin-like peptide 2, FBgn0036046, CG8167); *Ilp6* (Insulin-like peptide 6, FBgn0044047, CG14049); *InR* (Insulin-like receptor, FBgn0283499, CG18402); *Pdk1* (Pyruvate dehydrogenase kinase, FBgn0017558, CG8808); *Akt1* (Akt1, FBgn0010379, CG4006); *foxo* (forkhead box, sub-group O, FBgn0038197, CG3143); *G6P* (Glucose-6-Phosphatase, FBgn0031463, CG15400); *Pepck* (Phosphoenolpyruvate carboxykinase, FBgn0003067, CG17725); *GlyP* (Glycogen Phosphorylase, FBgn0004507, CG7254); *GlyS* (Glycogen Synthase, FBgn0266064, CG6904); *Ide* (Insulin degrading metalloproteinase, FBgn0001247, CG5517); *PyK* (Pyruvate kinase, FBgn0267385, CG7070); *PEK* (pancreatic eIF-2α kinase, FBgn0037327, CG2087); *Akh* (Adipokinetic hormone, FBgn0004552, CG1171); *ATGL* (*Bmm*, bummer- adipose triglyceride lipase, FBgn0036449, CG5295); *tgl* (tangled_Phosphatidic Acid Phospholipase A1- triglyceride lipase, FBgn0084120); *Keap1* (Kelch-like ECH-associated protein 1, FBgn0038475, CG3962); *Trxr-1* (Thioredoxin reductase-1, FBgn0020653, CG2151); *Rp49* (*RpL32*, Ribosomal protein L32, FBgn0002626, CG7939).

Tissues dissection, sorting of flies and haemolymph extraction

Proteasome is regulated in a tissue, sex and age-dependent manner [26]; thus, experiments were performed in dissected female or male somatic (head and thorax) tissues collected from young, middle-aged or old flies. For sorting experiments, female and male flies were anesthetized using CO_2 24 h prior to the experiment and the same number of individuals was used per sample. Haemolymph was isolated as described previously (Tsakiri et al., 2013).

Total RNA extraction and quantitative Real-Time PCR (Q-RT-PCR) analyses

Total RNA was extracted from flies' somatic tissues and converted to cDNA with the OneScript® cDNA Synthesis Kit of ABMGOOD (G234). cDNA was then subjected to Q-RT-PCR analysis using the HOT FIREPol® EvaGreen® qPCR Mix Plus of SOLISBIODYNE (08-24-00001) as described previously [21,33]. Primer sets were as described before [8,33,46].

Extraction of protein; immunoblot analyses and detection of protein carbonyl groups

Pooled or sex-sorted flies' somatic tissues or isolated mitochondria were homogenized on ice in Nonident P-40 (NP-40) lysis buffer [0.1 % Nonidet P-40, 150 mM NaCl, 50 mm Tris/HCl buffer (pH 8.0)] enriched with protease inhibitors and centrifuged for 10 min at 19,000 x g (4°C). The protein content of each sample was quantified by Bradford assay (Bio-Rad) and analyzed by SDS-PAGE/immunoblotting as described before [26,46]. Immunoblots were developed using an enhanced chemiluminescence Western blot detection kit (Bio-Rad Laboratories, Inc; 1705060S). For the detection of protein carbonyl groups, the OxyBlot protein oxidation detection kit (Millipore, Billerica, MA; #s7150) was used as per manufacturer's instructions. ImageJ was used to quantify protein expression in blots.

Native gel electrophoresis of proteasomes; measurement of ROS, cathepsin B, L and proteasome enzymatic activities

Proteasomes were analyzed according to Elsasser et al. (2005) with minor modifications. Somatic tissues from 20 young flies were collected in lysis buffer [50 mM Tris- HCl (pH 7.6), 5mM $MgCl_2$, 10% Glycerol, 5mM ATP and 1mM DTT]; tissues were homogenized, and samples were centrifuged 10 min at 9.000x g (4°C). Supernatants were collected, and the protein content of each sample was quantified using Bradford assay. Native-PAGE 3-12% gradient pre-cast Bis-Tris gels (Novex-Life Technologies, Thermo-Fisher Scientific)] were used to resolve 26S proteasomes. Prepared samples (50 μg of purified proteasomes) were mixed with 5x sample buffer [250 mM Tris-HCl (pH 7.4), 50% glycerol, 60 ng/ml xylene cyanol] just before loading. Electrophoresis was carried out at 4°C with an applied voltage of 100-110 Volts (~23-25 mA) for 3 h. Following completion of electrophoresis, gels were carefully transferred to a clear glass dish containing developing buffer [50 mM Tris-HCl (pH 7.4), 5 mM MgCl2, and 1 mM ATP]. To assess proteasome activity, gels were incubated with 50 μM of suc-LLVY-AMC, a fluorogenic substrate for CT-L peptidase activity, in developing buffer for 30 min at 37°C. Next, gels were exposed to UV and fluorescent bands were visualized by standard gel-imaging systems. For immunoblotting, remaining proteins were transferred to polyvinylidene fluoride membranes and immunoblotting was performed as described above. ROS levels, cathepsin and proteasome activities were measured as described previously [26,30, Tsakiri et al., 2013) and

expressed as (%) values *vs*. respective controls. When both male/female samples were analyzed equal numbers of male/female flies were used.

Mitochondria isolation, measurement of mitochondrial respiration, blue native-PAGE and measurement of GLU, TREH and GLY Levels

Mitochondria isolation, respiration analyses and blue native PAGE were performed as described before (Nijtmans et al., 2002; Ferguson et al., 2005; Cogliati et al., 2013); in most cases dissected somatic tissues from 30 flies were analyzed. GLU, GLY and TREH levels from indicated tissues were measured as described previously [46] (Barrio et al., 2014). At least 3 replicates per genotype or experimental condition were performed.

Adipose and muscle tissue preparation for immunohistochemistry and CLSM viewing

Adipose tissue was attached to the dorsal abdominal area and was isolated after removing head, thorax and the internal organs. Muscle tissue was recovered from the thoracic area after removing head and abdomen. Dissections were performed in PBS and tissues were fixed in 4 % formaldehyde for 15 min, washed in PBS containing 0.3 % Triton X-100 and were then incubated with the primary antibody (1:100) for 1 h in RT. Secondary antibodies (1:500), BODIPY (Molecular Probes™, TFS) (1:100), or DAPI (Thermo Fischer Scientific) staining were applied for 30 min in RT. Samples were washed in PBS, mounted in Mowiol® 4-88 (Sigma) and viewed at a Nikon C1 Confocal Laser Scanning Microscope (CLSM) equipped with a 40×, 1.0 NA differential interference contrast (DIC), 60×, 1.4 NA DIC Plan Apochromat objectives using the EZC1 acquisition and analysis software (Nikon). In most experiments 5-7 animals per population were analyzed. Measurement of CLSM stained structures (e.g. lipid droplets) was performed by ImageJ.

Larval fat bodies preparation for LysoTracker™ staining

Following dissection of 3rd instar stage larvae and removing all internal organs, fat bodies were attached to the dorsal abdominal area. Isolated fat bodies were simultaneously stained with DAPI and 100 μM LysoTracker Red (L-7528, Molecular Probes) as per manufacturer's instructions; samples were viewed using CLSM. The relative (%) quantification of the lysosomes per larval fat body area (μm^2) was performed using Image J.

Statistical Analysis

Statistical analysis of the results was performed as previously described [26]. Presented experiments were analyzed at least in duplicates, unless otherwise indicated. Data points correspond to the means of the independent experiments; error bars denote standard deviation (SD). For statistical analysis, MS Excel (Microsoft, Redmond, WA, USA) and the Statistical Package for Social Sciences (IBM

SPSS 19.0 for Windows; IBM, Armonk, NY, USA) were used. Significance at P< 0.05 or P< 0.01 is indicated in graphs by one or two asterisks, respectively.

Supplementary References

L. Barrio, A. Dekanty and M. Milán, "MicroRNA-mediated regulation of Dp53 in the *Drosophila* fat body contributes to metabolic adaptation to nutrient deprivation," *Cell Reports*, vol. 8, pp. 528-41, 2014.

S. Cogliati, C. Frezza, M.E. Soriano and et al., "Mitochondrial Cristae Shape Determines Respiratory Chain Supercomplexes Assembly and Respiratory Efficiency," *Cell*, vol. 155, no. 1, pp. 160-171, 2013.

S. Elsasser, M. Schmidt and D.B. Finley, "Characterization of the Proteasome Using Native Gel Electrophoresis," *Ubiquitin and Protein Degradation, Part A. Academic Press*, vol. 398, pp. 353–363, 2005.

M. Ferguson, M.J. Mockett, Y. Shen and et al., "Age-associated decline in mitochondrial respiration and electron transport in *Drosophila melanogaster*," *Biochemical Journal*, vol. 390, pp. 501–511, 2005.

L.G. Nijtmans, N.S. Henderson and I.J. Holt, "Blue Native electrophoresis to study mitochondrial and other protein complexes," *Methods*, vol. 26, pp.327–334, 2002.

E.N. Tsakiri, K.K. Iliaki, A. Höhn, S. Grimm, I.S. Papassideri, T. Grune and I.P. Trougakos, "Diet-derived advanced glycation end products or lipofuscin disrupts proteostasis and reduces life span in *Drosophila melanogaster*," *Free Radical Biology and Medicine*, vol. 65, pp. 1155–1163, 2013.

V.A. Vernace, L. Arnaud, T. Schmidt-Glenewinkel, M.E. Figueiredo-Pereira, "Aging perturbs 26S proteasome assembly in *Drosophila melanogaster*." *FASEB Journal*, vol. 21, pp 2672–2682, 2007.

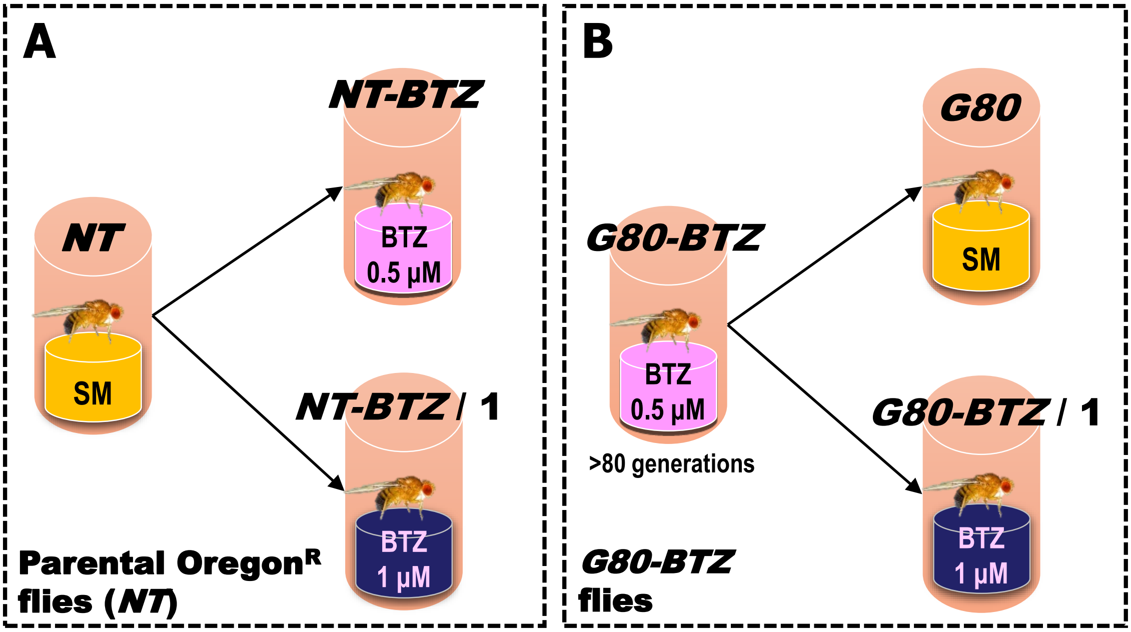

Nomenclature used for non-treated Oregon^R flies (NT) cultured in standard medium (SM) and *transiently* exposed to 0.5 or 1 µM BTZ

Nomenclature used for Oregon^R flies cultured in SM containing 0.5 µM BTZ for >80 generations (*G80-BTZ*) and *transiently* exposed to BTZ-free SM or to SM containing 1 µM BTZ

Manola et al. Fig. S1

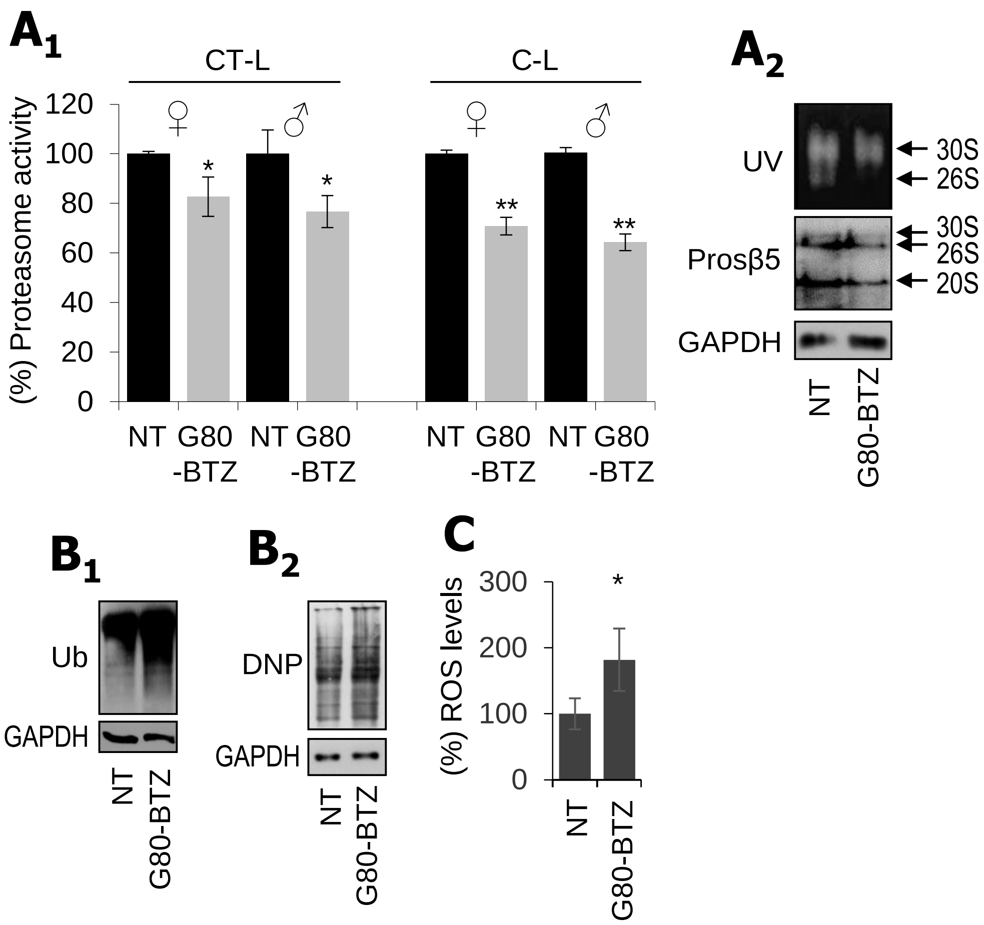

Manola et al. Fig. S2

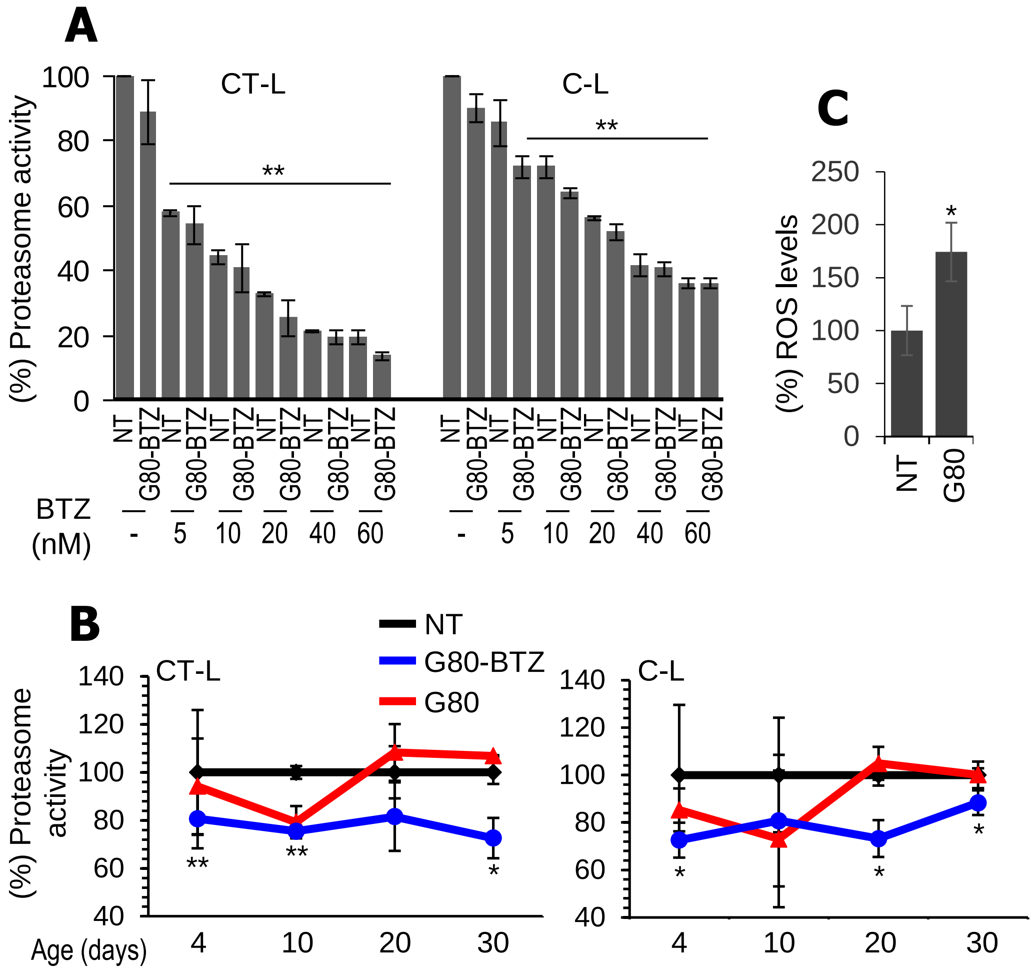

Manola et al. Fig. S3

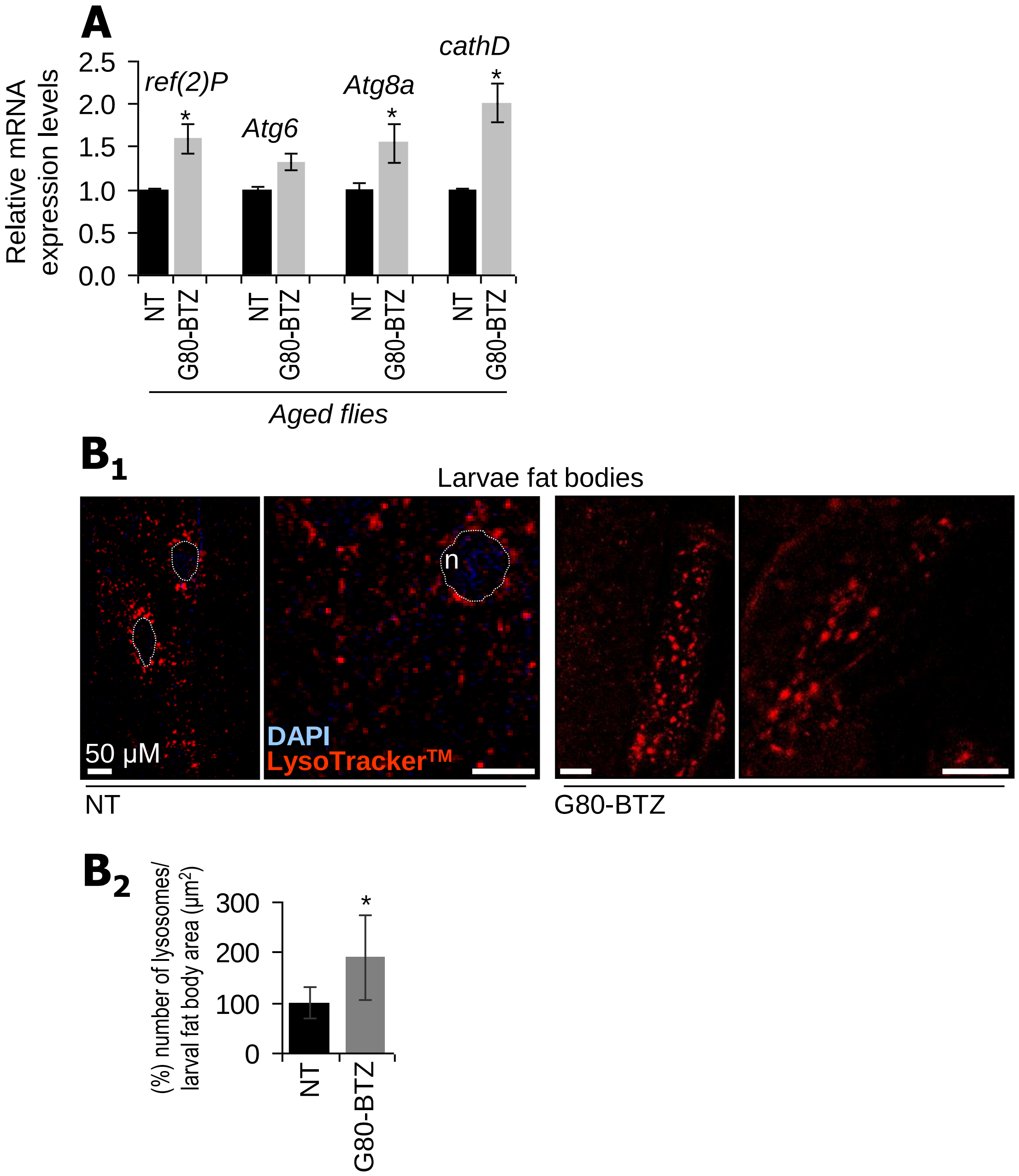

Manola et al. Fig. S4

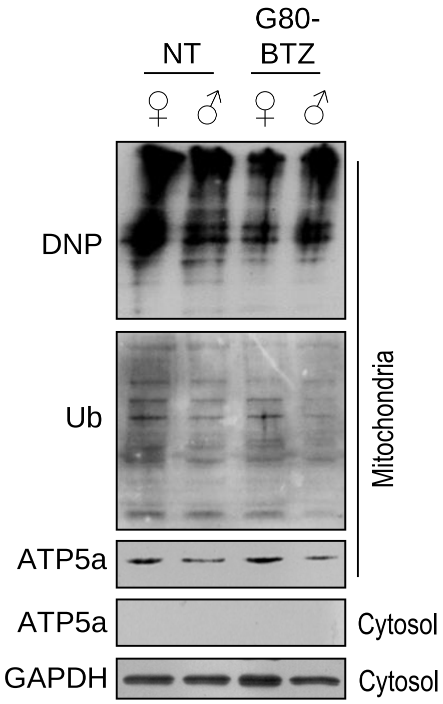

Manola et al. Fig. S5

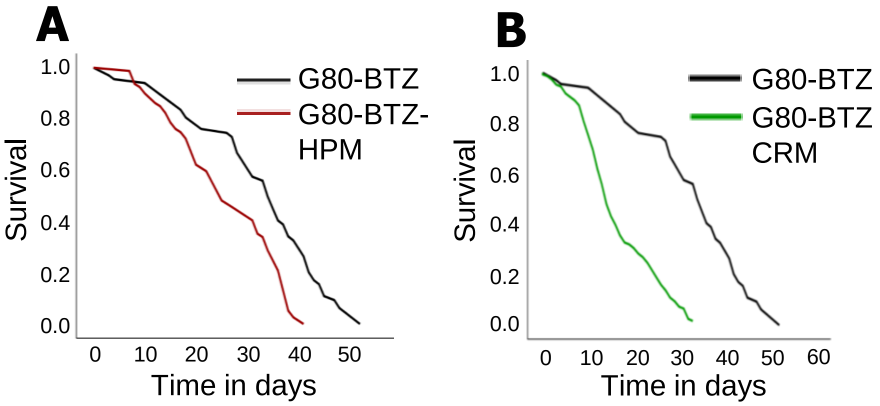

Manola et al. Fig. S6

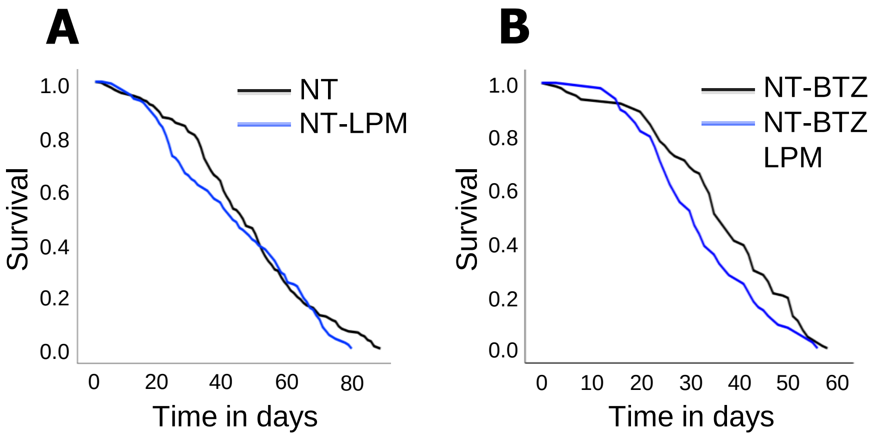

Manola et al. Fig. S7

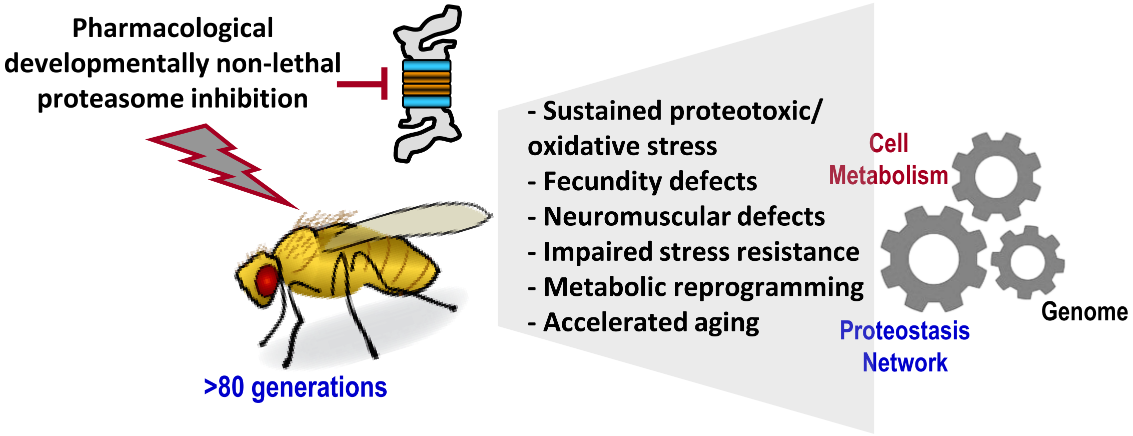

Manola et al. Graphical Abstract

Supplementary Table S1. Summary of lifespan experiments.

Figure	Sample	Mean Lifespan (LF) +/- s.e.m. (Days)		Median Lifespan +/- s.e.m. (Days)		% Median LF *vs* . control	Max (Days)	Log Rank P Value				
								NT ♀	G80-BTZ ♀	NT ♂	G80-BTZ ♂	*Total Animals Died/Total*
Fig. 1E$_1$	NT ♀	53.3	1.8	58	1.3	100	84		0.000	0.246	0.000	82/94
	G80-BTZ ♀	30.0	1.8	32	1.6	55	51	0.000		0.000	0.182	54/55
	NT ♂	49.8	1.9	50	2.9	86	84	0.246	0.000		0.000	82/93
	G80-BTZ ♂	27.0	1.4	27	0.9	47	51	0.000	0.182	0.000		86/90
								NT	G80	*Total Animals Died/Total*		
Fig. 1E$_2$	NT	46.4	1.3	46	2.1	100	89		0.000	226/269		
	G80	34.7	1.0	35	0.7	76	71	0.000		240/292		
								NT-BTZ / 1 ♀	G80-BTZ / 1 ♀	NT-BTZ / 1 ♂	G80-BTZ / 1 ♂	*Total Animals Died/Total*
Fig. 2D	NT-BTZ / 1 ♀	19.7	0.7	20	0.7	100	29		0.000	0.046	0.024	40/43
	G80-BTZ / 1 ♀	25.6	0.9	27	1.2	135	39	0.000		0.000	0.000	80/81
	NT-BTZ / 1 ♂	21.4	0.9	23	1.8	115	30	0.046	0.000		0.261	38/40
	G80-BTZ / 1 ♂	21.7	0.8	20	1.3	100	36	0.024	0.000	0.261		69/75
								NT ♀	NT ♀ - CQ	G80-BTZ ♀	G80-BTZ ♀ - CQ	*Total Animals Died/Total*
Fig. 3E$_1$	NT ♀	54.9	1.4	59	0.9	100	84		0.000	0.000	0.000	110/129
	NT ♀ - CQ	45.1	2.2	43	1.5	73	69	0.000		0.000	0.000	36/40
	G80-BTZ ♀	32.0	1.3	33	1.0	56	53	0.000	0.000		0.000	100/106
	G80-BTZ ♀ - CQ	16.9	1.0	16	0.8	27	27	0.000	0.000	0.000		34/38
								NT ♂	NT ♂ - CQ	G80-BTZ ♂	G80-BTZ ♂ - CQ	*Total Animals Died/Total*
Fig. 3E$_2$	NT ♂	48.6	1.5	50	2.1	100	75		0.000	0.000	0.000	113/130
	NT ♂ - CQ	38.2	2.0	37	3.1	74	64	0.000		0.000	0.000	39/39
	G80-BTZ ♂	27.1	1.2	27	0.8	54	51	0.000	0.000		0.000	122/129
	G80-BTZ ♂ - CQ	16.5	0.9	17	0.6	34	28	0.000	0.000	0.000		35/37
								G80-BTZ	G80-BTZ-LPM	*Total Animals Died/Total*		
Fig. 6A$_1$	G80-BTZ	29.5	2.6	30	4.7	100	52		0.304	31/34		
	G80-BTZ-LPM	34.7	2.1	36	3.7	120	59	0.304		35/36		
								G80-BTZ	G80-BTZ-HPM	*Total Animals Died/Total*		
Fig. S6A	G80-BTZ	32.1	1.5	34	1.2	100	52		0.000	65/68		
	G80-BTZ-HPM	25.6	1.2	25	2.9	74	41	0.000		78/80		
								G80-BTZ	G80-BTZ-CRM	*Total Animals Died/Total*		
Fig. S6B	G80-BTZ*	32.1	1.5	34	1.2	100	52		0.000	65/68		
	G80-BTZ-CRM	16.4	0.7	14	0.7	41	33	0.000		139 / 148		
								NT	NT-LPM	*Total Animals Died/Total*		
Fig. S7A	NT	46.0	1.3	46	2.2	100	89		0.095	226/250		
	NT-LPM	43.0	1.8	42	3.1	91	80	0.095		138/142		
								NT-BTZ	NT-BTZ-LPM	*Total Animals Died/Total*		
Fig. S7B	NT-BTZ	35.8	1.2	36	1.2	100	58		0.06	121/149		
	NT-BTZ-LPM	31.9	1.2	31	1.7	86	56	0.06		93/104		

** shown also in S6A*
